# Prenatal Substance Exposure Is Associated with Increased Placental *DRD1* Dopamine Receptor Gene Expression and Striatal Gray Matter Volume in Children

**DOI:** 10.3390/genes17080958

**Published:** 2026-08-15

**Authors:** Tara E. Samson, Donato DeIngeniis, Maki S. Koyama, Roberto Bailey, Claire J. Brabander, Ariadna Cilleros Portet, Corina Lesseur, Ahmed Duke Shereen, Yoko Nomura

**Affiliations:** 1 The Graduate Center and Queens College, City University of New York, New York, NY 11367, USA; tsamson@gc.cuny.edu (T.E.S.); donato.deingeniis@qc.cuny.edu (D.D.);; 2 Hunter College Campus Schools, New York, NY 10128, USA; robertobailey@hunterschools.org; 3 Department of Environmental Medicine, Icahn School of Medicine at Mount Sinai, New York, NY 10029, USA; 4 Advanced Science Research Center, The Graduate Center, City University of New York, New York, NY 10031, USA

**Keywords:** prenatal substance exposure, placental gene expression, *DRD1*, dopaminergic system, striatum, gray-matter volume, hormesis, Developmental Origins of Health and Disease

## Abstract

Background/Objectives: Prenatal substance exposure (PSE) to alcohol, cannabis, and other psychoactive drugs affects over 500,000 pregnancies annually in the US and is consistently associated with adverse birth and childhood outcomes, but the underlying biological mechanisms are not well-understood. Given the dopaminergic system’s role in substance use and child development, this study aimed to examine and connect the effects of PSE on dopaminergic placental gene expression at birth and on striatal brain volumes in middle childhood. We hypothesized that PSE would lead to reduced dopamine receptor D1 (*DRD1*) gene expression and smaller striatal gray-matter volumes (GMVs) and that placental *DRD1* gene expression would be positively associated with striatal GMV. Methods: PSE, placental gene expression, and T1-weighted MRI data were drawn from a pilot study (*n* = 34) within the longitudinal cohort Stress in Pregnancy study. PSE was defined by any amount of alcohol, tobacco, or cannabis use during pregnancy. Results: Unexpectedly, children with PSE had a trend toward increased placental *DRD1* gene expression (β = 0.497, *p* = 0.056) and significantly larger GMV in the right putamen (β = 0.374, *p* = 0.018) and nucleus accumbens (NAc) (β = 0.389, *p* = 0.029) than unexposed children. Independent of PSE, higher placental *DRD1* gene expression at birth was also associated with larger right NAc GMV (β = 0.617, *p* = 0.015) in middle childhood. Conclusions: These findings contrast prior cross-sectional work linking high-dosage PSE to smaller striatal volumes and inconsistent patterns of dopaminergic gene expression, suggesting potential compensatory placental mechanisms reflected in biological outcomes across child development.

## 1. Introduction

In the United States, nearly 14% of pregnant people used psychoactive drugs or alcohol while pregnant in 2021, resulting in approximately 500,000 infant births that year with prenatal substance exposure (PSE) [1]. PSE is consistently associated with a range of negative health outcomes, including birth complications like reduced gestational age, lower birth weight, and more frequent neonatal infections [2,3]. As the infant ages, PSE continues to confer adverse developmental effects such as poor motor performance in toddlerhood [4], elevated problem behavior trajectories through early childhood [5], and lower cognitive abilities in adolescence [6]. Moreover, PSE is associated with alterations in the brain, including reduced subcortical volumes, disrupted white matter maturation, and widespread disruptions in functional connectivity during infancy [7], which in turn are linked to heightened risk for developmental delays [7,8]. While the adverse consequences of PSE are clear, the biological pathways linking PSE to neural and developmental alterations in childhood remain poorly understood.

The Developmental Origins of Health and Disease (DOHaD) theory provides a framework for understanding these pathways. This DOHaD framework [9,10] posits that factors in the perinatal environment can impact fetal and later life health. Perinatal factors are proposed to alter the starting calibrations of biological systems by modifying fetal gene expression through epigenetic mechanisms in anticipation of a presumed stressful future environment. These predictive adaptations may or may not be well suited to the world the fetus eventually inhabits, and mismatches between the predicted and actual world can ultimately contribute to lifelong adverse health outcomes. The placenta, an organ that develops alongside the fetus in utero and serves as the interface between maternal and fetal circulation and the regulator of the fetal environment, is an ideal place to observe these starting calibrations because it is naturally shed at birth and shares the child’s DNA [11]. In support of the DOHaD theory, a growing body of research demonstrates that the impacts of prenatal stressors (i.e., maternal smoking, air pollution, chemicals) on infant health outcomes are associated with and mediated by alterations in placental epigenetics [12,13,14].

Emerging literature also indicates that PSE may alter placental epigenetics via distinct biological mechanisms [15,16,17]. When alcohol, nicotine, and/or cannabis are used during pregnancy, their chemical byproducts cross from the mother’s blood to the fetus through the placenta [3] and cause oxidative stress through the accumulation of toxic molecules and misfolded proteins [16,17,18]. Oxidative stress in the placenta has been identified as a key driver of epigenetic modifications and disrupted fetal growth [19]. Importantly, a moderate level of oxidative stress in the placenta is normative in fetal development, and the placenta naturally generates antioxidants to keep oxidative stress under control [20]. However, chronic exposure to toxicity, as in many cases of PSE, is thought to overwhelm this adaptive response and contribute to adverse developmental outcomes.

The biological impacts of PSE described above may specifically alter gene expression in the dopaminergic system, which is implicated in both substance use and child motor and emotional development [21,22]. Prior research supports that prenatal exposure to alcohol is associated with increased methylation of promoter regions of the dopamine transporter gene (*DAT*) in cord blood DNA of babies whose mothers engaged in binge drinking during pregnancy compared to non-binge drinking controls [23]. Prenatal cannabis exposure has also been linked to decreased gene expression of dopamine receptor D2 (*DRD2*) in the fetal nucleus accumbens in a rodent model [24]. Less is known about the effects of PSE on the dopamine receptor D1, which plays distinct roles in reward processing and motor function [22]. Animal research provides some evidence linking PSE to changes in dopamine signaling, particularly in striatal brain regions where dopamine receptor D1s are primarily expressed [25], though directionality is mixed. In one study, rats with prenatal exposure to cannabis showed increased excitability of dopamine neurons projecting to the striatum compared to unexposed controls [26]. Other studies demonstrated mice with in utero exposure to cocaine had reduced D1 receptor protein expression in the embryonic striatum [27] and enhanced D1 receptor signaling in the striatum in adulthood [28]. The mixed directionality of these results and limitations of animal models motivate further study to explore how the effects of PSE manifest in human dopaminergic systems at distinct levels of exposure and timepoints in development.

In the brain, the striatum serves as the principal interface between dopaminergic signaling and cortico-subcortical circuitry [29]. The striatum is therefore a dense site of D1 expressing circuitry and may be particularly sensitive to alterations in placental dopaminergic signaling during pregnancy. Within the striatum, the putamen, caudate, and nucleus accumbens (NAc) each represent a functionally distinct node of the dopaminergic circuit [29,30,31,32]. The putamen receives modulatory dopaminergic signals from the substantia nigra via D1 receptors to initiate movement and motor habit learning in the nigrostriatal pathway [30]. The caudate receives similar input but is more behaviorally implicated in the planning and execution of complex, goal-directed actions [31]. Finally, the NAc is the primary terminal of the mesolimbic pathway, originating in the ventral tegmental area and rich with D1 receptors. The NAc is behaviorally implicated in reward learning, motivation, and addiction [32]. Prior research indicates that PSE is associated with smaller striatal volumes in childhood, namely in the NAc and putamen following polysubstance use [33] and in the NAc and caudate following tobacco use [34], though the biological mechanisms linking PSE to alterations in striatal volume remain unclear. It also remains to be seen whether these PSE-related neural alterations in childhood can be traced back to variations in placental gene expression.

Notably, low levels of PSE may not confer the same biological stress and subsequent adverse developmental effects as higher levels of exposure. A large meta-analysis found that light-to-moderate alcohol exposure in utero did not significantly alter genome-wide DNA methylation in offspring [35]. Furthermore, low doses of THC (the main psychoactive molecule of cannabis) have been shown to improve placental cell viability and mitochondrial function by acting as an antioxidant to reduce oxidative stress [36]. In this same study, however, the effect was reversed at high doses of THC, which were found to increase oxidative stress and placental cell death [36]. This work demonstrates a potential dual effect of cannabis exposure on fetal development and suggests that low levels of exposure may even elicit adaptive responses (i.e., hormesis). The concept of hormesis [37], wherein low levels of tolerable stress lead to positive developmental outcomes while high levels of toxic stress lead to adverse outcomes, is well-developed in the adverse childhood experiences (ACEs) literature [38,39]. Collectively, this literature suggests that low levels of PSE may elicit compensatory biological responses whose developmental consequences remain to be determined.

Together, this research illustrates that PSE may affect both the dopaminergic system and the developing striatum through epigenetic modifications to dopamine receptor expression. However, to our knowledge, no study has yet examined the developmental associations between PSE, placental dopamine receptor D1 (*DRD1*) gene expression, and childhood striatal gray matter volume (GMV) within a human cohort. Understanding these associations could illuminate how prenatal exposures to drugs and alcohol relate to placental programming and ultimately childhood neurological consequences. Such findings may also inform scientific understanding of the directionality and developmental timing of PSE-related outcomes. The present study aims to examine the associations between PSE, placental *DRD1* gene expression, and childhood striatal GMV using a pilot sample drawn from an ongoing birth cohort project—the Stress in Pregnancy (SIP) study [40]. While behavioral consequences of PSE are well-documented, this study focuses on the upstream molecular and structural correlates of PSE—placental *DRD1* gene expression and childhood striatal volume—as a foundation for later work connecting these markers to behavioral outcomes. Based on the literature reviewed above, we hypothesized that prenatal substance exposure would be associated with (1) lower placental *DRD1* expression and (2) smaller striatal GMV and that (3) *DRD1* expression would be positively associated with striatal GMV.

## 2. Methods

*Participants and Procedures*

Thirty-four mother–child dyads were enrolled in a pilot study for the ongoing longitudinal SIP study [40]. The full SIP study investigates the effects of prenatal stressors, particularly in utero exposure to the 2012 Superstorm Sandy, on child developmental trajectories. Pregnant women were recruited from prenatal clinics in New York City during their second trimester of pregnancy between 2010 and 2015. Consent was obtained in line with Institutional Review Board guidelines at the City University of New York, New York-Presbyterian/Queens, and the Icahn School of Medicine at Mount Sinai. Pregnant mothers completed psychosocial assessments during pregnancy. Following delivery, biological samples, including a placenta sample for gene expression quantification, were collected. Mothers and children completed annual longitudinal follow-ups, and prior studies have investigated relationships between prenatal exposures and child emotional, behavioral, and neurologic outcomes in this sample [41,42,43,44]. In this pilot study follow-up, a subset of children (*n* = 34, 70.6% female, mean age = 8.7 years) from the SIP study also completed one MRI scan. Participant demographics are further detailed in Table 1.

*Measures*

*Prenatal Substance Exposure*. During their second trimester of pregnancy, mothers completed a study-specific checklist assessing tobacco, alcohol, and cannabis use during pregnancy, as well as the Structured Clinical Interview for DSM-IV-TR (SCID) [45] to assess substance use disorders. Prenatal substance exposure (*n* = 12, 35.3%) was defined by any maternal endorsement of tobacco, alcohol, and/or cannabis use during pregnancy. Of the 12 mothers endorsing PSE, seven reported tobacco use, 10 reported cannabis use, and one reported alcohol use, with considerable overlap across substances.

*DRD1 expression.* Placental biopsies, free of maternal decidua, were collected and processed from the fetal side of the placenta, which is derived from the fetal genome and contains mixed cell types (i.e., trophoblasts, stromal fibroblasts, endothelial cells), consistent with standard bulk-tissue approaches used in placental gene expression research [46,47]. *DRD1* gene expression was quantified using a custom-designed panel on the nCounter Platform (NanoString Technologies, Seattle, WA, USA) including 40 candidate imprinted, HPA and neurodevelopment genes selected a priori [48]. Counts were normalized with the NanoString Norm R package against the geometric mean of spike-in controls to account for hybridization and recovery differences. Sample content was standardized with the geometric mean of housekeeping genes (*GAPDH, RPL19*, and *RPLP0*). Values below the background limit of detection (LOD) for each sample (mean ±2SD of negative control probes) were set at the value of the LOD divided by the square root of two to maintain sample variability. Genes with values above the background level in >50% of the samples were considered unexpressed. *DRD1* was expressed beyond the limit of detection in >50% of the original cohort study sample [48]. For this secondary analysis, only *DRD1* was examined from the full genetic panel due to its theorized relevance to PSE and the striatum. In this pilot study sample, half of the mothers (*n* = 17) had provided placenta samples at birth, including 29.4% (*n* = 5) with PSE. Demographics of the sample with both gene expression and MRI data were similar to those of the full MRI pilot sample (Table 2) and did not significantly differ by PSE status (Table 3).

*Striatal Volume*. Participants underwent MRI scans on a Siemens 3T MRI scanner, including a standard high-resolution structural T1-weighted sequence (inversion time/repetition time/echo time = 1070/2500/2.9 ms, flip angle =  8.0 degrees, field of view = 256 mm, matrix = 256 × 256, slices = 176, resolution = 1mm isotropic). Real-time motion detection and correction were implemented using Volumetric Navigators [49]. Raw imaging data were preprocessed, and subcortical gray matter volumes (GMVs) were calculated using the FreeSurfer pipeline [version 7.4.1] with the automated subcortical segmentation (aseg) procedure [50]. To account for individual differences in head size, which may be directly affected by prenatal substance exposures [51], all GMV estimates were normalized by total intracranial volume, yielding proportional GMV values.

Six striatal regions of interest (ROIs) were defined a priori in the left and right caudate, putamen, and nucleus accumbens due to the hypothesized sensitivity of the dopaminergic system to PSE. Given prior evidence of lateralized striatal effects in developmental populations with PSE [7], left- and right-hemisphere ROIs were separately examined to allow detection of hemisphere-specific associations.

*Covariates*

All analyses were controlled for the child’s sex because placental epigenetics and gray matter volume have been shown to be influenced by sex [52,53]. Additionally, analyses involving striatal volumes were controlled for age at MRI assessment and prenatal Superstorm Sandy exposure, as these are established correlates of gray matter volume in children [49] and particularly within this sample [41].

*Statistical Analysis*

Linear regression in IBM SPSS Statistics (version 29; IBM Corporation, Armonk, NY, USA) was used to test effects of PSE on placental *DRD1* expression, PSE on striatal GMVs, and *DRD1* expression on striatal GMVs. Following hypothesis testing and based on preliminary results, an exploratory moderation analysis was performed to test whether the association between *DRD1* expression and striatal GMV differed by PSE status. Given the pilot nature of this study and the a priori selection of ROIs based on established dopaminergic circuitry, corrections for multiple comparisons were not applied [54]. All analyses are treated as exploratory and hypothesis-generating; effect sizes *(*β) and 95% confidence intervals are reported to facilitate interpretation independent of p-value thresholds. Data visualization was performed in R Studio (version 1.4.1717; R Core Team) with ggplot2.

## 3. Results

**Figure 1:**
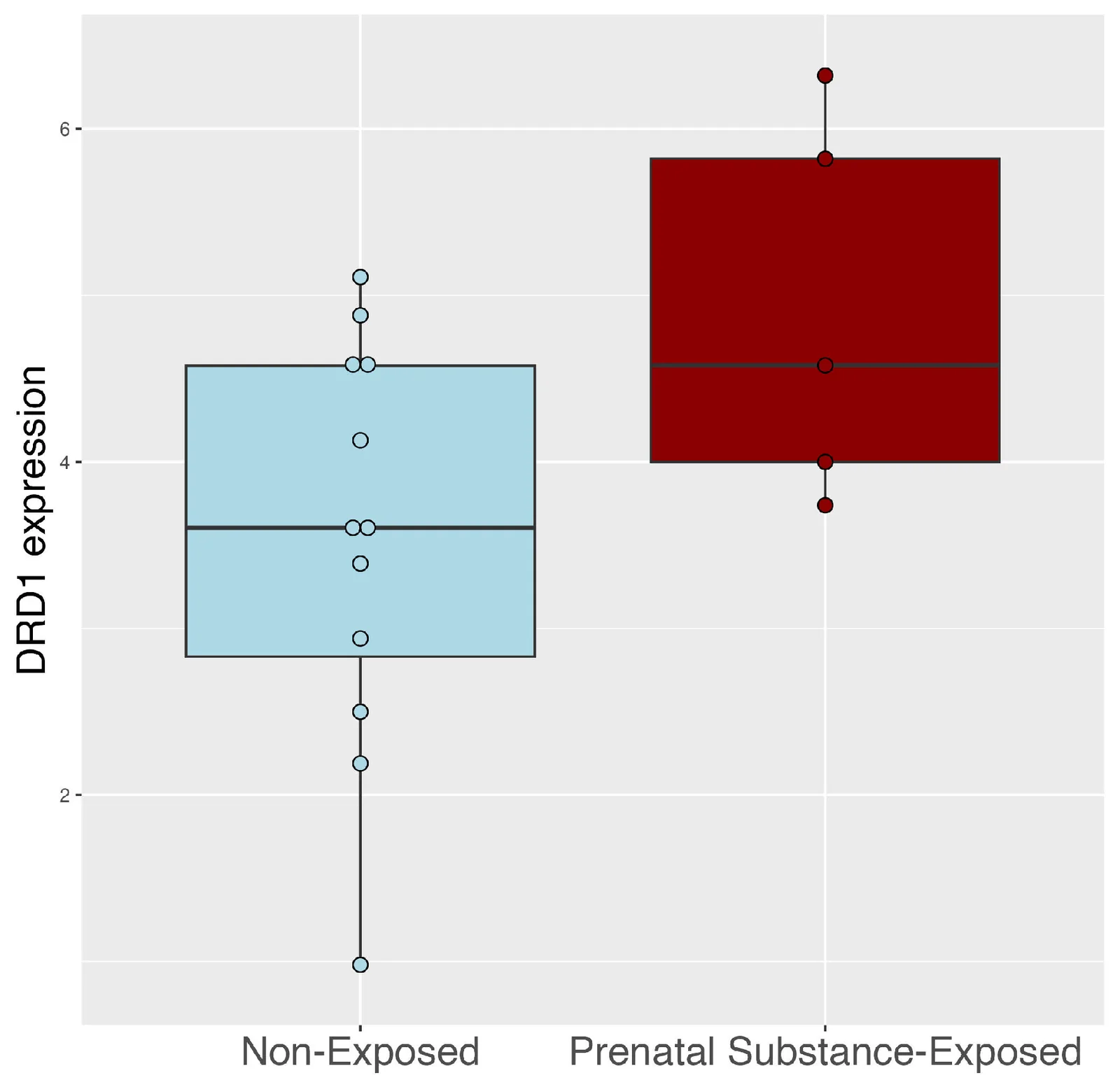
Box plot of placental dopamine receptor D1 (*DRD1*) gene expression for non-exposed (*n* = 12) and prenatal substance-exposed (*n* = 5) groups. Solid middle lines represent group medians. Lower and upper hinges correspond to the first and third quartiles. Dots represent individual participant scores. *DRD1* expression values are unadjusted (raw).

As seen in Table 4, children with PSE exhibited higher mean placental *DRD1* gene expression (mean (*M*) = 4.89, *SD* = 1.13) than non-exposed children (*M* = 3.54, *SD* = 1.23), with this difference reaching trend level significance (β = 0.497, *p* = 0.056) when controlling for child sex. Figure 1 further displays the pattern of group differences using unadjusted values.

**Figure 2:**
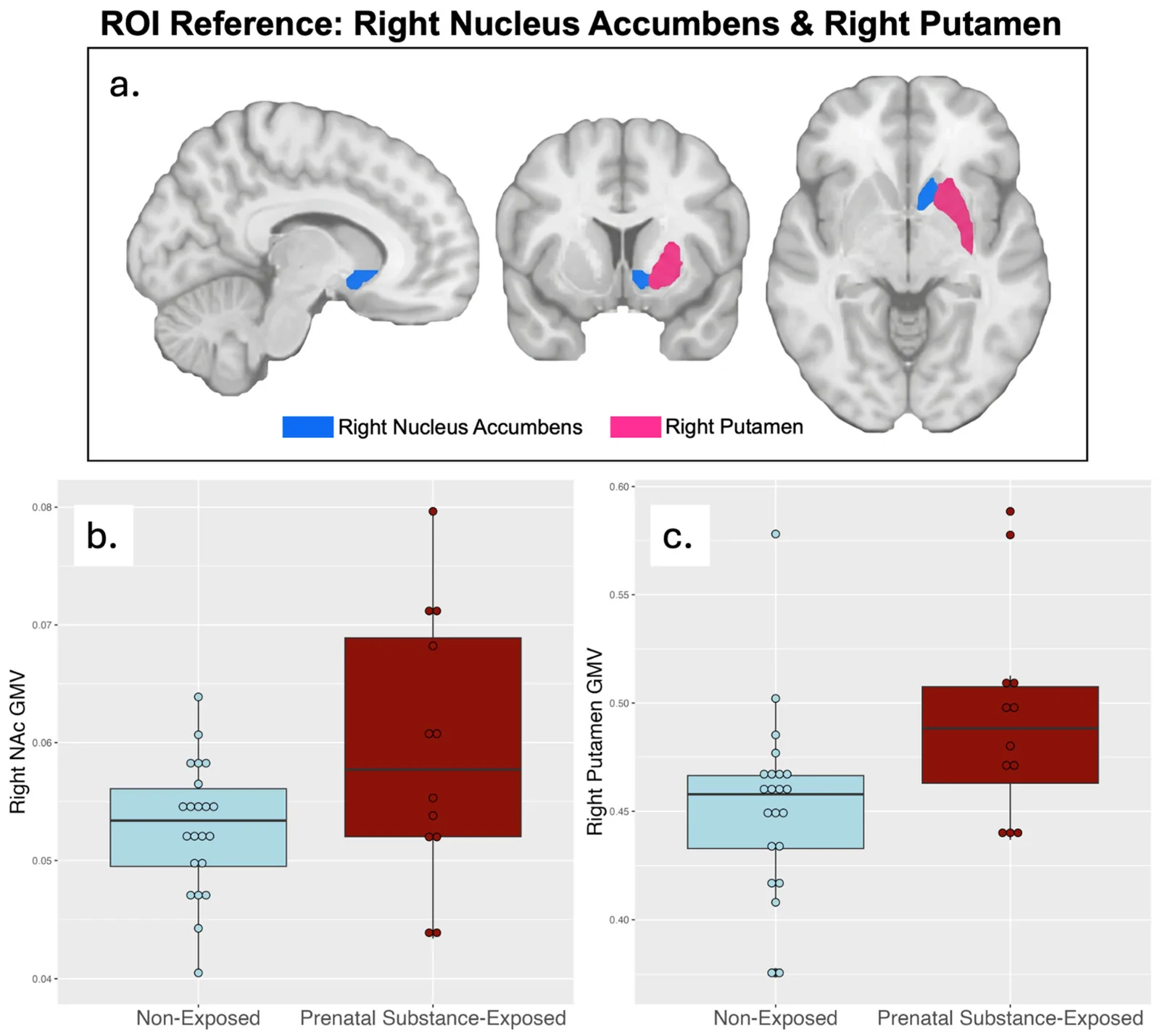
(**a**) Nucleus accumbens (NAc) (blue) and right putamen (pink) shown on a standard template to illustrate anatomical location. Box plots of (**b**) right NAc and (**c**) right putamen gray-matter volume (GMV) for non-exposed (*n* = 22) and prenatal substance-exposed (*n* = 12) groups. GMV values are unadjusted (raw).

Additionally, children with PSE exhibited significantly larger GMV in the right NAc (*M* = 0.059, *SD* = 0.011) than non-exposed children (*M* = 0.053, *SD* = 0.006) (β = 0.389, *p* = 0.029) (Figure 2b), controlling for child sex, age at MRI, and Sandy exposure. The same pattern was found for right putamen GMV (β = 0.374, *p* = 0.018). Children with PSE exhibited significantly larger right putamen GMVs (*M* = 0.49, *SD* = 0.049) than non-exposed children (*M* = 0.45, *SD* = 0.043) (Figure 2c), controlling for child sex, age at MRI, and Sandy exposure. No significant group differences were observed in the caudate or left hemisphere regions.

**Figure 3:**
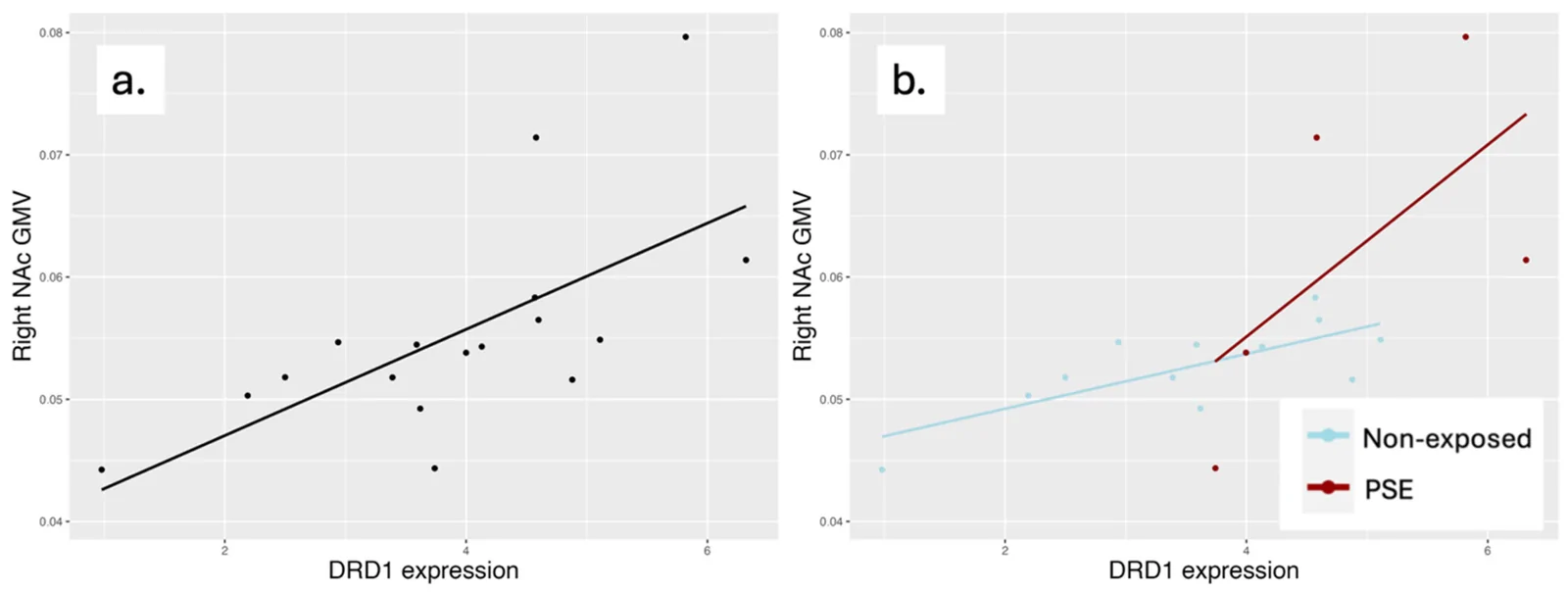
Scatterplots of the association between placental dopamine receptor D1 (*DRD1*) gene expression and right nucleus accumbens (NAc) gray-matter volume (GMV) as (**a**) the full sample and (**b**) stratified by non-exposed and prenatal substance-exposed (PSE) status. GMV values are unadjusted (raw).

Table 5 and Figure 3 show the main effect and interaction effect of *DRD1* expression with PSE on right NAc GMV. As the table shows, placental *DRD1* expression at birth was positively associated with right NAc GMV in middle childhood (β = 0.617, *p* = 0.015) (Figure 3a), controlling for child sex, age at MRI, and Sandy exposure. While the interaction was not statistically significant (β = 1.37, *p* = 0.207), inspection of the interaction pattern showed that high placental DRD1 expression appeared to be associated with larger right NAc GMVs among children with PSE than among non-exposed children (Figure 3b). Placental *DRD1* expression was not significantly associated with right putamen, caudate, or left hemisphere regions.

## 4. Discussion

This pilot study provides novel and preliminary evidence linking PSE to increased placental expression of the dopamine receptor D1 gene, *DRD1*, and larger GMV in the right NAc and right putamen in middle childhood, contrary to our initial hypotheses. We observed a large effect of PSE on increased *DRD1* expression as well as moderate effects of PSE on increased striatal GMV in the right putamen and right NAc. Findings also indicate a positive and large effect of placental *DRD1* expression on right NAc GMV in childhood, connecting placental dopaminergic signaling to childhood striatal development, though these effect size estimates should be interpreted cautiously given the small sample size.

Our first finding of an association between PSE and increased placental expression of *DRD1* contrasts with prior research conducted in mice [27]. In addition to species differences, Kubrusly and Bhide examined fetal striatal tissue, whereas we measured placental expression, which may account for divergent findings given the tissue-specific nature of gene expression. The dopamine system serves different functions across these tissues: receptor-mediated neuronal signaling in the striatum [55] versus monoamine transport, clearance, and endocrine regulation in the placenta [56]. These tissue-specific regulatory mechanisms may therefore produce divergent expression patterns in response to the same prenatal exposure. Nevertheless, evidence of fetal programming [9,10] and the placenta–brain axis [57] suggest placental epigenetics are important indicators of prenatal conditions and can predict neurodevelopmental outcomes [12,13,14]. Substance type may further contribute to the divergent findings. While Kubrusly and Bhide examined cocaine exposure, our PSE group was defined by alcohol, tobacco, and/or cannabis use, which have been shown to differ from cocaine in their effects on dopaminergic signaling [58].

Similarly, our second finding of an association between PSE and larger GMVs in the right putamen and NAc contrasts with prior research that showed children with PSE to have smaller right putamen and NAc volumes than non-exposed children [33,34,59]. Striatal volumes show considerable inter-individual variability in middle childhood [60], and both the present study and Walhovd and colleagues’ relied on relatively small sample sizes; the opposing directions of effect may therefore partly reflect sampling variability rather than a true biological divergence. This possibility could be supported through testing in larger samples. Notably, 71% of the PSE in the study by Walhovd and her colleagues was to opiates/heroin, and all exposed children were adopted or in foster care, while all children in the present study lived with their birth mother through age at MRI. This variance in postnatal environment may also account for our divergent findings. Interpreting our first two results together, we highlight that the developmental consequences of PSE likely differ by type of substance used as well as by the postnatal environment.

Given the literature reviewed above [35,36] showing contrasting effects of low versus high levels of PSE on child outcomes, we theorize that our observed effects may represent hormesis, an adaptive biological response to a tolerable level of stress [37,61]. Because we defined PSE as any amount of endorsed substance use during pregnancy, including single-substance and light use, our sample may represent lower levels of exposure than those characterized in prior research. This hypothesis remains speculative, as our study did not stratify by level of PSE and therefore cannot confirm a dose-dependent hormetic relationship. In line with the diverging behavioral outcomes observed following low versus high levels of early life stress in the ACEs literature [39], our participants may represent cases of positive stress effectively managed by placental buffering capacity, while contrasting literature appears to demonstrate the maladaptive effects of high levels of PSE representing toxic stress under harsher circumstances such as foster care [33] or regular binge drinking during pregnancy [23]. To further support this hypothesis, future work should incorporate quantification of direct measures of biological stress to test the proposed diverging effects of positive versus toxic stress.

On the molecular level, we propose that low levels of PSE may introduce a tolerable degree of oxidative stress to the placental environment. The placenta is biologically adapted to manage oxidative stress by generating corresponding antioxidants, and moderate levels of oxidative stress have been shown to support healthy placental function and precise developmental timing [20]. Moreover, the majority of our sample with PSE (83%) reported cannabis use, and emerging research suggests that low levels of THC can also act as an antioxidant to support placental viability and functioning [36], though at high concentrations, the effect reverses from supportive to damaging. This pattern of supported homeostasis at low doses and reversed effects at high doses parallels our observed effects of PSE on *DRD1* expression and striatal volume, which run counter to previously observed effects at high doses of PSE [27,33]. These diverging outcomes may occur through alterations to DNA methylation or growth factors, particularly within the highly PSE-relevant dopaminergic system. In parallel clinical work, some studies have shown that recreational cannabis use during pregnancy does not impact risk for neurodevelopmental disorders, such as autism spectrum disorder (ASD), in offspring [62,63]; however, cannabis use disorder (i.e., high volume, high frequency use) during pregnancy does lead to elevated risk of ASD [64]. To further develop the emerging patterns detected in this preliminary work and directly test the proposed biological mechanisms, future studies with adequately powered samples should stratify effects by PSE level and drug type.

Our third finding that placental *DRD1* expression is associated with right NAc volume in middle childhood highlights the interconnectedness of PSE and the dopaminergic system throughout fetal and child development. This finding is aligned with prior research linking genetic polymorphisms in *DRD1* to ventral striatum reactivity in adolescence [21]. Our result linking *DRD1* placental expression at birth to striatal volume in middle childhood may represent an earlier developmental correlate of Baker et al.’s observations. This finding supports the DOHaD hypothesis, demonstrating placental dopaminergic programming reflected in child brain volume over eight years later, underscoring how prenatal factors can have lasting structural consequences throughout development. To our knowledge, this is among the first studies to examine whether PSE moderates the association between *DRD1* expression and striatal volume. Although the *DRD1* × PSE interaction did not reach statistical significance (*p* = 0.207), the association between *DRD1* expression and right NAc GMV appeared visually stronger among children with PSE than among unexposed children (Figure 3b). Given the small subsample with placental expression data and PSE (*n* = 5), this pattern is strictly exploratory and warrants replication in larger samples. One speculative interpretation based in the DOHaD framework is that PSE may amplify placental dopaminergic programming with downstream consequences for striatal development.

*Limitations and Future Directions*

There are several limitations to this work worth acknowledging. First, the statistical power of this pilot study is limited by its relatively small sample size, which may lead to unstable or inflated estimates. Our relatively small sample size also did not provide sufficient power to compare outcomes across type and amount of substance use or polysubstance use. Importantly, however, the detected moderate to large effect sizes suggest trending associations may become statistically significant in adequately powered samples. Second, because PSE was defined dichotomously, our measure could not capture dose, frequency, or timing of exposure, meaning individuals with any endorsed use were grouped together. This may have biased our findings by either attenuating true dose-dependent effects or inflating group differences driven by heavier-exposure individuals. While we suspect our sample primarily represents low severity PSE, we cannot distinguish between these possibilities with the current measure. Future work with larger and less imbalanced samples should incorporate measures of frequency, amount, and timing of exposure to clarify dose-response relationships. Third, our sample, particularly the group with PSE, was disproportionately Hispanic/Latino, reflecting the demographics of our recruitment sites. While we did not expect participant ethnicity to influence results, this imbalance should be considered when interpreting results and may have reduced generalizability to other populations. Fourth, our sample, particularly the group with PSE, was also disproportionately female. While we controlled for sex in all analyses, our sample size is underpowered to rule out potential sex differences, which should be considered in future work with larger samples. Fifth, our reliance on self-reported substance use is a limitation because self-reports have been shown to underestimate rates of substance use assessed via toxicology [65,66], including among pregnant populations [67], although self-reports are generally more accurate for less stigmatized substances [68], including those examined in this study. Sixth, our findings should also be interpreted with appropriate caution because we did not correct for multiple comparisons across striatal regions in this pilot study.

Given the opposite directionality of our findings compared to prior literature, further studies comparing effects between type, amount of substance exposure, and timing of outcome measurement (e.g., at birth vs. middle childhood vs. adolescence) could provide additional insight. Future studies could also benefit from considering the heritability of substance use vulnerability by comparing *DRD1* expression levels between mother and child to clarify whether group differences reflect novel compensatory responses in the child or inherited patterns. Finally, future work should examine whether PSE-related variations in *DRD1* and corresponding right NAc volume mediate downstream behavioral outcomes such as impulsivity or substance use initiation that have been independently linked to both PSE and striatal volume [69,70,71,72].

## 5. Conclusions

Overall, our data reinforce that the placenta serves as an active and sensitive genetic interface between maternal cues, such as PSE, and child development. We posit that the unexpected increases in placental *DRD1* expression and striatal GMV may reflect adaptive or compensatory responses to low doses of tolerable PSE-related stress. Replication in larger cohorts is needed to clarify whether the observed neurobiological effects of PSE are linked to adaptive or maladaptive behavioral outcomes and to formally test moderation effects. This work advances understanding of the neurobiological mechanisms underlying associations between prenatal substance exposure and child neurodevelopment and may inform prevention efforts and targeted interventions for at-risk children. Ultimately, these findings underscore that the developmental consequences of PSE are both pervasive and adaptable within complex systems of human biological risk and resilience.

## Figures and Tables

**Table 1:** Demographic characteristics of the total MRI pilot sample and subsets with and without prenatal substance exposure (PSE).

	Total Sample	PSE	No PSE	Statistics	*p*
	N = 34	N = 12	N = 22		
**Maternal Race,** N (%)					
White	1 (2.9%)	0 (0%)	1 (4.5%)	*χ*^2^ (3) = 1.37	0.712
Black	6 (17.6%)	2 (16.7%)	4 (18.2%)		
Hispanic/Latino	22 (64.7%)	9 (75.0%)	13 (59.1%)		
Mixed/Other	5 (14.7%)	1 (8.3%)	4 (18.2%)		
**Child Sex,** N (%)					
Female	24 (70.6%)	10 (83.3%)	14 (63.6%)	*χ*^2^ (1) = 1.45	0.228
Male	10 (29.4%)	2 (16.7%)	8 (36.4%)		
**Child Age at MRI**					
Mean (SD)	8.7 (1.9)	8.7 (2.1)	8.7 (1.9)	*t* (32) = −0.087	0.931

**Table 2:** Demographics of the full MRI pilot sample and the subset with placental gene expression data.

	Full Sample	Subset with Placenta	Statistics	*p*
	N = 34	N = 17		
**Substance use**				
N (%)	12 (35.3%)	5 (29.4%)	*χ*^2^ (1) = 0.52	0.473
**Maternal Race,** N (%)				
White	1 (2.9%)	0	*χ*^2^ (3) = 3.47	0.325
Black	6 (17.6%)	2 (11.8%)		
Hispanic/Latino	22 (64.7%)	11 (64.7%)		
Mixed/Other	5 (14.7%)	4 (23.5%)		
**Child Sex,** N (%)				
Female	24 (70.6%)	11 (64.7%)	*χ*^2^ (1) = 0.57	0.452
Male	10 (29.4%)	6 (35.3%)		
**Child Age at MRI**				
Mean (SD)	8.7 (1.9)	8.7 (1.6)	*t* (32) = −0.042	0.967

**Table 3:** Demographics of the subset with placental gene expression data with and without PSE.

	Subset with Placenta	Placenta with PSE	Placenta Without PSE	Statistics	*p*
	N = 17	N = 5	N = 12		
**Maternal Race,** N (%)					
Black	2 (11.8%)	0	2 (16.7%)	*χ*^2^ (2) = 1.13	0.569
Hispanic/Latino	11 (64.7%)	4 (80.0%)	7 (58.3%)		
Mixed/Other	4 (23.5%)	1 (20.0%)	3 (25.0%)		
**Child Sex,** N (%)					
Female	11 (64.7%)	4 (80.0%)	7 (58.3%)	*χ*^2^ (1) = 0.73	0.394
Male	6 (35.3%)	1 (20.0%)	5 (41.7%)		
**Child Age at MRI**					
Mean (SD)	8.7 (1.6)	7.6 (1.2)	9.2 (1.6)	*t* (15) = 1.93	0.073

**Table 4:** Effects of prenatal substance exposure (PSE) on placental *DRD1* expression, controlling for child sex.

Variable	*b*	*SE*	95% CI	*p*
PSE	1.41	0.68	[−0.04, 2.85]	0.056

**Table 5:** Multiple linear regression of *DRD1*, PSE, and their interaction on right NAc GMV.

Variable	Model 1 *b*	*SE*	95% CI	*p*	Model 2 *b*	*SE*	95% CI	*p*
*DRD1* exp.	0.004	0.001	[0.001, 0.01]	0.015	0.002	0.002	[−0.002, 0.01]	0.25
PSE	—	—	—	—	−0.02	0.02	[−0.06, 0.02]	0.36
PSE × *DRD1*	—	—	—	—	0.01	0.004	[−0.003, 0.01]	0.21

NB: Model 1 is the main effect-only model, and Model 2 includes an interaction term. Both models controlled for child sex, age at MRI, and prenatal Superstorm Sandy exposure.

## Data Availability

Data is available upon request to the corresponding author Y.N.
